# Topographic Dependence of Cropland Transformation in China during the First Decade of the 21st Century

**DOI:** 10.1155/2013/303685

**Published:** 2013-10-22

**Authors:** Yuejiao Li, Xiaohuan Yang, Wenli Long

**Affiliations:** ^1^State Key Laboratory of Resources and Environmental Information System, Institute of Geographic Sciences and Natural Resources Research, Chinese Academy of Sciences, Beijing 100101, China; ^2^University of Chinese Academy of Sciences, Beijing 100049, China; ^3^Zhejiang Academy of Agricultural Sciences, Zhejiang 310021, China

## Abstract

Terrain plays a critical role in the selection of cropland. As a physical and geographical element of the landscape, terrain is an important limiting factor in land use change and has a strong influence on human activities. The objectives of this study are to investigate the topographic characteristics of cropland-related transformations in China during the first decade of the 21st century and to explore the implications of land use change as it relates to securing a national food supply. A 2010 satellite-based land use dataset and the DEM data were used to conduct spatial statistical analysis using altitude, slope, and fragmentation data. The results showed the following. (1) As the urbanization and industrialization of China occur, and China attempts to replace this occupied cropland with newly reclaimed cropland, the topography of the most recently reclaimed cropland has been more poorly suited to farming than the topography of the occupied cropland it replaces in most provinces. (2) The area of occupied cropland was much larger than of those reclaimed; the qualities of occupied and reclaimed cropland were significantly different. (3) Land reclamation mainly occurred in northern China, instead of in southern China, which has a higher level of economic development. Our findings imply that the potential area available for cropland production may be limited.

## 1. Introduction

China has a large population on a relatively small amount of land, which creates a concern for the government regarding the consistent availability of an adequate amount of food [1]. In recent years, a series of policies designed to control land use have been repeatedly stressed by China's Ministry of Land and Resources. Unlike other countries where urbanized land is simply lost, China deals with land use change by attempting to replace farmland lost with newly reclaimed cropland by tracking land use at the province level. That is, these policies are designed to ensure that every province takes measures to actively promote and enforce regulations related to land use in a way that compensates for the occupation of cropland with the creation of new farmland elsewhere. The term “land reclamation” is itself a misnomer since, for example, wetlands are actually lost when they are “reclaimed” as farmlands. This reclaimed cropland is tracked at the province level in an attempt to supply each province and the country with a continuous supply of cropland and an adequate amount of food. As such, reclaimed cropland can also be thought of as compensatory cropland, compensating the province for cropland lost. While each of these terms may have unique shades of meaning, reclaimed cropland and compensation cropland all refer to the same areas of land. Occupied cropland refers to cropland that has been occupied for other uses. These land polices are also intended to ensure that the reclaimed croplands are of high quality, and this type of policy is important for guaranteeing a secure supply of food for the country. 

 China has complex terrain conditions that are very different between occupied cropland and the reclaimed cropland that replaces it; if these reclaimed lands are not managed appropriately, a serious crisis will occur, which will aggravate problems related to the capacity of the nation to produce an adequate supply of grain. Therefore, it is very important to compare the spatiotemporal characteristics of occupied croplands and the reclaimed croplands that replace them using topographical elements to develop appropriate methods for ecologically sound development of cropland resources and grain production [2, 3].

 Cropland occupation and reclamation analysis involves a temporal and spatial study of supply and demand for cropland productivity. It involves analyzing the demand for primary products and the need to sustainably regulate the quantity, quality, and use of cropland [4]. Several researchers have recently conducted analyses related to the occupation of cropland and compensation for lost cropland; these include analyses cropland occupation and cropland reclamation [5–14], countermeasures that can balance of occupation with reclamation [10, 14–18], evaluations of policies [19, 20], analyses of environmental impacts [21], suggestions for improving policies and mechanisms [22–25], and monitoring cropland needs and alerting policy makers of gross cropland imbalances [26]. An important issue that may have been ignored is the relationship between the patterns of cropland occupation and cropland reclamation and their influences combined with the influence of terrain factors.

 In the last half century, many researchers have studied the relationship between various types of terrain factors and the dynamic spatiotemporal patterns of cropland [27–29, 29–49]. The size of croplands increases with altitude [50]. With an increase in altitude, the ratio of cropland area to area of all land use types decreases [51] and the value of agricultural output per unit area also declines [52]. A similar relationship exists between the slope and the pattern of cropland, with croplands on steeper slopes requiring additional area to produce the same output. In addition, some topographic indices have been applied to describe the relationships between cropland distribution, cropland grading, and terrain. Wei et al. [53] determined that terrain factors not only impact the spatial patterns of cropland, but also affect the quality of cropland. According to the above studies, the quality of cropland is inversely proportional to two topographic factors, slope and elevation (i.e., croplands in flatter areas at low elevations are of higher quality).

 In cases where the total area of cropland remains unchanged, if the reclaimed cropland has worse terrain conditions compared with the occupied cropland, the equilibrium would be broken, resulting in serious negative impacts on primary productivity; that is, the balance between the amount of cropland in use and the amount of food produced will be disrupted with less food produced on the same number of hectares of land because the reclaimed cropland is less productive than the occupied cropland. Using topographic data and land use/land cover change data from 2000 to 2010, we analyzed the terrain features of China's occupied and reclaimed cropland using elevation, slope, and degree of fragmentation as the main analytical parameters. We also explored the significance of cropland occupation analysis for maintaining a secure food supply in China. Three important issues must be addressed: (1) the spatial characteristics of occupied and reclaimed cropland; (2) the analysis of the relationship between occupied cropland, reclaimed cropland and topographic factors; and (3) the impacts of the analysis of occupied and reclaimed cropland on land resources and the security of China's food supply.

## 2. Materials and Methods

### 2.1. Materials

Cropland change data and terrain data were acquired from the Data Center for Resources and Environmental Sciences, Chinese Academy of Sciences. Data with 1 km spatial resolution from three periods, 2000–2005, 2005–2008, and 2008–2010, included information such as the current land use types including occupied cropland and the source of land used to reclaim land for use as cropland. The original 1 : 100,000 land use data were extracted from remote sensing information from Landsat TM/ETM. Small noncultivated ground objects were not excluded from the size of cropland, so the area was considered a gross value. Slope data were extracted from terrain data. DEM (digital elevation model) data were obtained from the SRTM (Shuttle Radar Topography Mission) data.

### 2.2. Methods

Slope was divided into four levels, 0–5°, 5–15°, 15–25°, and >25° [50–53] (Table 1). The elevation was classified as <100 m, 100–200 m, 200–800 m, and >800 m.

 Spatial overlay analysis was performed using 1 km resolution cropland change data combined with the reclassification of the DEM (digital elevation model) data and slope information. Spatial statistics were compiled by province. Fragmentation analysis [34, 35] has mainly investigated the integrity of cropland mass. Having a relatively intact and large-scale cropland mass is usually important for agricultural productivity. Compared with broken plots, relatively intact large-scale cropland can make better use of the natural fertility of cultivated land. The fragmentation index used in landscape ecology was adopted for measuring cropland fragmentation (see (1)):

(1) $$\begin{array}{c} C=\frac{T}{A}, \end{array}$$

where *T* refers to cropland patch number and *A* refers to cropland area. Fragmentation values (*C*) range from 0 to 1, with the higher value indicating a more broken distribution of cropland resources. The fragmentation index was calculated using Fragstats (version 3.3) landscape ecology software. In addition, the rates of cropland occupation and reclamation at a particular point in time were used to analyze the characteristics of cropland occupation and reclamation; K-Means Cluster in SPSS was used to conduct cluster analysis of the rate of cropland occupation and the rate of cropland reclamation.

 Some of the areas were not suitable for large-scale cropland reclamation because of the terrain. Using only the spatial extent of the area of cropland being occupied and reclaimed cannot accurately reflect the actual situation of cropland occupation and reclamation. Therefore, the cropland occupation rate and cropland reclamation rate were used in the analysis.

 The cropland occupation and reclamation rates in a province from 2000 to 2010 were calculated using (2) and (3), respectively. Consider

(2) $$\begin{array}{c} \text{O}{\text{C}}_{j}=\frac{\sum_{j=2000}^{2010}O_{j}}{A_{2000}}, \end{array}$$

where OC_*j*_ refers to the cropland occupation rate in a province from 2000 to 2010, ∑_*j*=2000_^2010^ *O*_*j*_ refers to the area of cropland occupied in a province from 2000 to 2010, and *A*_2000_ refers to the total cropland area in a province in 2000. Consider

(3) $$\begin{array}{c} \text{S}{\text{U}}_{j}=\frac{\sum_{j=2000}^{2010}S_{j}}{A_{2000}}, \end{array}$$

where SU_*j*_ refers to the cropland reclamation rate in a province from 2000 to 2010, ∑_*j*=2000_^2010^ *S*_*j*_ refers to the area of cropland reclaimed in a province from 2000 to 2010, and *A*_2000_ refers to the cropland area in a province in 2000.

## 3. Analysis and Discussion

### 3.1. Temporal and Spatial Distribution of Cropland Occupation and Reclamation

**Figure 1:**
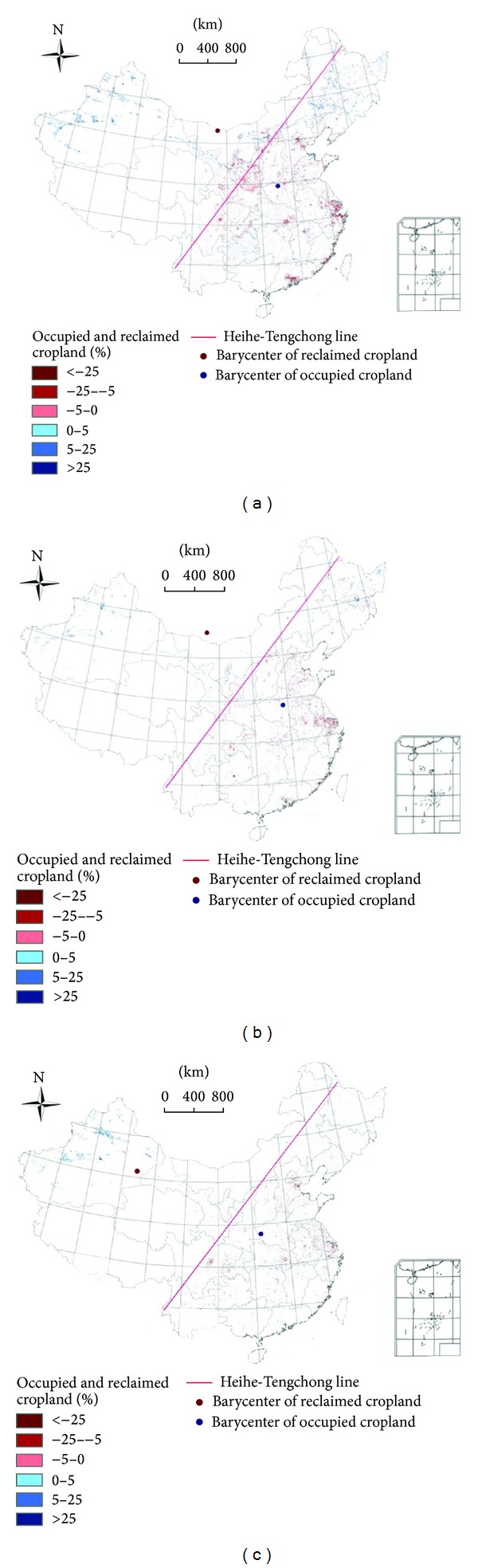
(a) Spatial distribution of occupied and reclaimed cropland in China from 2000 to 2005, (b) spatial distribution of occupied and reclaimed cropland in China from 2005 to 2008, and (c) spatial distribution of occupied and reclaimed cropland in China from 2008 to 2010.

An initial analysis of cropland patterns showed that most occupied cropland and most areas of reclaimed cropland were in southern China (Figures 1(a), 1(b), and 1(c)). This is important for identifying the distribution patterns of occupied and reclaimed cropland along what is called the Heihe-Tengchong Line. Heihe-Tengchong Line separates the more occupied cropland of eastern China from the more reclaimed cropland of western China. 

 The total area of occupied cropland was 451.88 × 10^4^ hm^2^ from 2000 to 2010 in China. The area of occupied cropland declined from 256.65 × 10^4^ hm^2^ to 117.95 × 10^4^ hm^2^ and finally to 83.05 × 10^4^ hm^2^ during 2000–2005, 2005–2008, and 2008–2010, respectively, accounting for 56.80%, 26.10%, and 18.38% of the total area, respectively (Table 2). That is, the total area of occupied cropland was gradually declining every year.

 The area of reclaimed cropland was 315.94 × 10^4^ hm^2^ from 2000 to 2010 in China. The area of reclaimed cropland declined from 198.37 × 10^4^ hm^2^ in 2000 to 65.28 × 10^4^ hm^2^ in 2005 and finally to 57.57 × 10^4^ hm^2^ in 2010 and accounted for 62.79% in 2000, 20.66% in 2005, and 18.22% in 2010 of the total area of available cropland, respectively. The area of reclaimed cropland was also gradually declining every year.

 Cropland occupation was most intensive from 2000 to 2005, mainly concentrated in the regions of Beijing-Tianjin-Tangshan, the Yangtze River Delta, the lower Pearl River Basin, Changsha-Zhuzhou-Xiangtan, Sichuan Basin, Guanzhong in Shaanxi Province, and northern Shaanxi. The rate of cropland occupation decreased slightly from 2005 to 2008, while cropland occupation of the Yangtze River Delta region was still very heavy. Compared with the first two time periods, the least amount of cropland was occupied from 2008 to 2010, while the Beijing-Tianjin-Tangshan and the Yangtze Delta Urban agglomerations continued to be the main areas of cropland occupation. In addition, the loss of cropland to the central Wuhan Urban agglomerations and the regions surrounding Chengdu and Chongqing were also very prominent.

**Figure 2:**
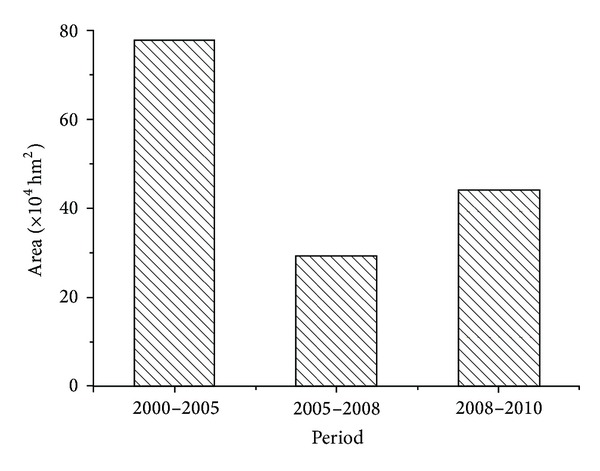
Reclaimed cropland area during different time periods in Xinjiang.

 The occupied cropland was mainly concentrated in northern China and accounted for 180.33 × 10^4^ hm^2^. This was mainly distributed in 15 provinces, particularly in regions such as the Songnen Plain, Chifeng in Inner Mongolia, near Ordos and Baotou, the Hexi Corridor region in Gansu Province, the Tianshan Mountains, and the middle and lower reaches of the Tarim River. In contrast, the spatial extent of reclaimed cropland was only 18.04 × 10^4^ hm^2^ in 16 southern provinces, an amount equal to only one-tenth of occupied cropland in northern China. In southern China, the reclaimed cropland was mainly distributed in Guizhou and Jiangxi Provinces, with a scattered and spotty distribution pattern in Guizhou and in the vicinity of the Poyang Lake region in Jiangxi. The main regions for cropland reclamation were in the Xinjiang region and in Gansu Province. The amount of cropland reclamation decreased considerably from 2005 to 2008, but the area of reclamation was still mainly located in the northern regions of Fuyuan County and Mudanjiang in Heilongjiang Province, the Chifeng region of Inner Mongolia, the Ningxia Loop area, the Hexi Corridor of Gansu Province, the Xinjiang Tianshan region, and the Tarim River Basin. From 2008 to 2010, the area of reclaimed cropland continued to decrease in every province, but the areas of reclaimed cropland in the Xinjiang region increased slightly (Figure 2).

 Based on the distribution of reclaimed and occupied cropland in China, most of the occupied cropland was distributed to the east of the Heihe-Tengchong line, and most of the reclaimed cropland was to the west. The center of gravity of occupied cropland can be thought of as a geographic location at the center of where urbanization is occurring in China; this center shifted a little during the three time periods studied, and all were in Henan Province. In contrast, the center of gravity of reclaimed cropland shifted considerably. From 2000 to 2005, it was in Inner Mongolia near the border with China; then, it shifted northward to a point slightly north of the Chinese border in south central Mongolia, from 2005 to 2008, and later moved to the Xinjiang autonomous region by 2010. The movement of the center of gravity of reclaimed cropland was mainly caused by a significant decrease in the spatial extent of reclaimed cropland in northeastern China and in Gansu Province.

 The area of reclaimed cropland was larger than for occupied cropland in many provinces and regions, such as Xinjiang, Heilongjiang, Inner Mongolia, Jilin, and Qinghai, which had reclaimed cropland areas of 131.69 × 10^4^ hm^2^, 21.69 × 10^4^ hm^2^, 15.63 × 10^4^ hm^2^, 4.85 × 10^4^ hm^2^, and 0.54 × 10^4^ hm^2^, respectively. The cropland area decreased in some developed provinces. For example, the area for actively used cropland declined 37.05 × 10^4^ hm^2^ in Jiangsu, 24.63 × 10^4^ hm^2^ in Guangdong, 24.54 × 10^4^ hm^2^ in Zhejiang, 21.24 × 10^4^ hm^2^ in Shandong, 21.18 × 10^4^ hm^2^ in Shaanxi, and 20.53 × 10^4^ hm^2^ in Sichuan. The reclaimed cropland was distributed mainly in northern China, and the occupied cropland was mostly in southern China or Shandong Province.

 Based on the clustering results (Table 3), for the reclaimed cropland, only Xinjiang was in the first group with a reclamation rate of 24.93%; the second group had five provinces and regions, including Heilongjiang, Inner Mongolia, Ningxia, Qinghai, and Gansu; and the remaining regions were in the third group. For the cropland occupation rate, only Shanghai was in the first group; Beijing, Tianjin, Ningxia, Jiangsu, Zhejiang, Fujian, and Guangdong were in the second group; and the remaining regions were in the third group.

### 3.2. The Relationship between Occupied and Reclaimed Cropland and Slope

Occupied cropland was significantly positively associated with slopes of 0–5° and the percentage of an occupied cropland area decreased with the increasing slope.

**Figure 3:**
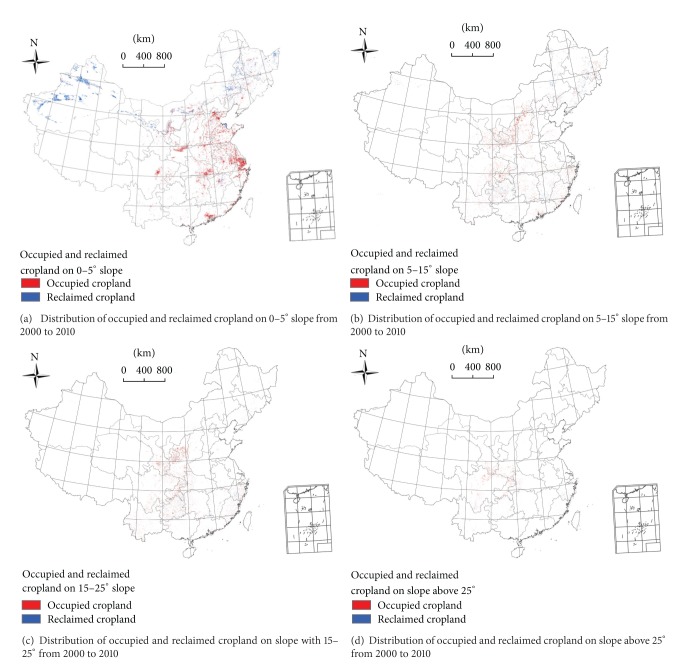
Distribution of occupied and reclaimed cropland on different slope segments from 2000 to 2010.

 Five provinces in northern China, all of which were poorly developed provinces, showed an obvious favorable trend in reclaimed cropland, in which more land was being reclaimed than occupied and the reclaimed cropland had slopes that were favorable for use in agriculture (Figures 3(a), 3(b), 3(c), and 3(d)). First, the area of land with various slopes used for cropland increased in Xinjiang, Heilongjiang, and Jilin Provinces from 2000 to 2010. Second, the slope of occupied cropland was above 25° and below 25° on reclaimed cropland in Inner Mongolia. The occupied area in Inner Mongolia was much less than the area of reclaimed cropland. Third, most of Qinghai's occupied cropland had slopes of >5°, while reclaimed cropland had slopes of <5°. That is, most of the area of reclaimed cropland in Inner Mongolia had slopes favorable to agriculture (<5°) and this amount was greater than the area of occupied area where occupation mostly occurred on land with slopes of >5°. 

 Throughout China, the extent of cropland occupation exceeded the rate of cropland reclamation from 2000 to 2010, and the area of land reclamation was inadequate and far less than what is needed. In some provinces with an adequate amount of cropland, occupied cropland often had slopes of 0–5° which were favorable to agriculture, while reclaimed cropland had slopes of >5°, which were less favorable for agricultural use. The spatial extent of cropland on different slopes all decreased in 24 provinces and regions, including Hebei, Beijing, Tianjin, Liaoning, Ningxia, Shandong, Shanxi, Shaanxi, Henan, Jiangsu, Tibet, Shanghai, Anhui, Chongqing, Hubei, Zhejiang, Sichuan, Guizhou, Hunan, Fujian, Yunnan, Guangxi, Guangdong, and Hainan. In these provinces and regions, the occupied cropland area with 0–5° slope declined by more than 10 × 10^4^ hm^2^ in nine provinces: Hebei, Shandong, Henan, Jiangsu, Anhui, Hubei, Zhejiang, Sichuan, and Guangdong. It declined between 1–10 × 10^4^ hm^2^ in 13 provinces and regions: Beijing, Tianjin, Liaoning, Ningxia, Shaanxi, Shanxi, Shanghai, Chongqing, Hunan, Fujian, Yunnan, Guangxi, and Hainan. It only declined by less than 1 × 10^4^ hm^2^ in Tibet and Guizhou. Most of the reclaimed cropland had slopes of >5° in Jiangxi Province and the amount of cropland being reclaimed was less than that being occupied; occupied cropland in Jiangxi had slopes of <5°. The intensity of efforts to protect cropland needs to be increased. Large areas of cropland with ideal terrain conditions had been affected by urbanization, especially in the provinces mentioned above.

 The average slope of occupied cropland was greater than the average slope of all cropland in seven provinces. On the contrary, 25 provinces had a lower average slope on reclaimed cropland compared with the slope of all croplands in each province. We also found that the average slope of reclaimed cropland was lower than the slope of occupied cropland in 18 provinces. Statistical analysis indicated that the average slope of reclaimed cropland in China was 0.99°. The average slope in reclaimed cropland was 1.08°, 0.91°, and 0.68° in 2000–2005, 2005–2008, and 2008–2010, respectively. The average slope of reclaimed cropland in China gradually decreased over time (Table 4).

### 3.3. The Relationship between Occupied and Reclaimed Cropland and Altitude

**Figure 4:**
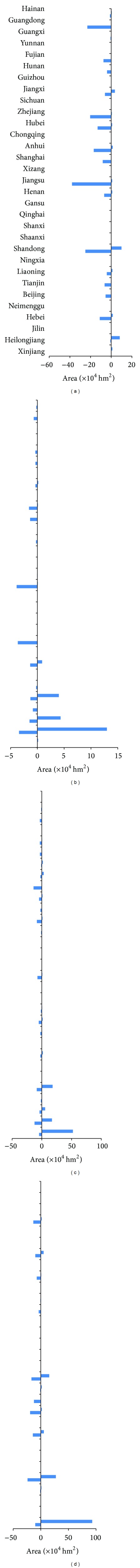
Distribution of occupied and reclaimed cropland at different altitudes in all provinces.

Figures 4(a), 4(b), 4(c), and 4(d) correspond to the area of occupied and reclaimed cropland at altitudes of <100 m, 100–200 m, 200–800 m, and >800 m, respectively. The area of occupied cropland below an altitude of 100 m was significantly greater than the area at other elevations (Figure 4). The area of low altitude occupied cropland was considerable for most provinces, such as Hebei, Shandong, Jiangsu, Anhui, Hubei, Zhejiang, and Guangdong. These provinces accounted for more than 10 × 10^4^ hm^2^. That is, most occupied cropland in China was in favorable terrain conditions at altitudes of <100 m, while cropland was rarely reclaimed at this altitude range.

 Compared with altitudes of <100 m, less occupied and reclaimed cropland existed at altitudes of 100–200 m. The area of occupied and reclaimed cropland in each province was never more than 5 × 10^4^ hm^2^. The area of reclaimed cropland was also no more than 5 × 10^4^ hm^2^; only in Heilongjiang did the extent of reclaimed cropland exceed 10 × 10^4^ hm^2^. In the 200–800 m altitude range, there was more than 10 × 10^4^ hm^2^ of occupied cropland in Sichuan and Heilongjiang. At the same altitude range, there was 52.20 × 10^4^ hm^2^ of reclaimed cropland in Xinjiang, which accounted for 16.52% of the total reclaimed cropland in China. Occupied and reclaimed croplands were observed at altitudes of >800 m. There was about 93.08 × 10^4^ hm^2^ reclaimed cropland in Xinjiang, accounting for 29.46% of the total reclaimed cropland in China; 27.21 × 10^4^ hm^2^ reclaimed cropland was present in Inner Mongolia and 15.25 × 10^4^ hm^2^ was in Gansu.

 The average elevation of occupied cropland was greater than the average elevation of all cropland in six provinces. On the contrary, 21 provinces had a lower average elevation of reclaimed cropland than that for all cropland in each province. We also found that the average elevation of reclaimed cropland was lower than the average elevation of occupied cropland in 15 provinces (Table 5).

### 3.4. Fragmentation Analysis of Occupied and Reclaimed Cropland

**Figure 5:**
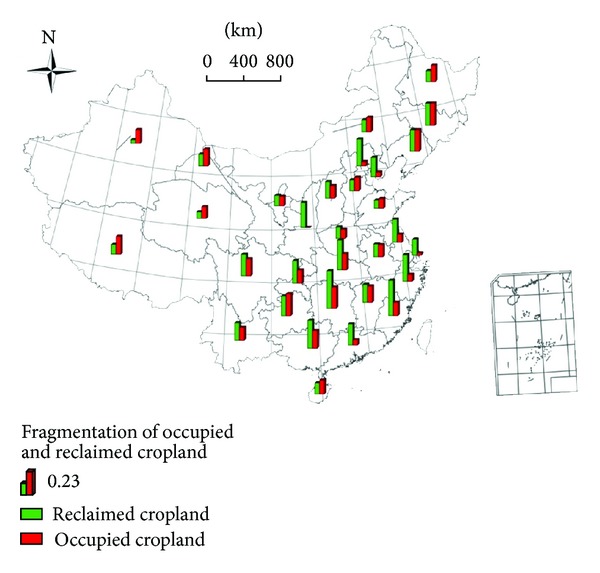
Distribution of occupied and reclaimed cropland in all provinces (excluding Hong Kong, Macau, and Taiwan) in China from 2000 to 2010.

Only 10 provinces, Xinjiang, Heilongjiang, Hebei, Inner Mongolia, Shandong, Qinghai, Gansu, Tibet, Guizhou, and Hainan, had fragmentation of reclaimed cropland that was less than the fragmentation of occupied cropland (Figure 5). In most other provinces, reclaimed cropland occurred in small scattered plots, but occupied cropland was relatively intact in large-scale, unfragmented pieces of land. The fragmentation of occupied cropland in descending order is Jilin (0.27), Guizhou (0.26), Liaoning (0.25), Hunan (0.25), Tibet (0.21), Guangxi (0.21), Sichuan (0.20), Gansu (0.20), Jiangxi (0.20), Heilongjiang (0.19), Hubei (0.19), Inner Mongolia (0.17), Hainan (0.17), Xinjiang (0.16), Fujian (0.16), Hebei (0.16), Chongqing (0.16), Yunnan (0.15), Shanxi (0.15), Anhui (0.14), Qinghai (0.14), Shandong (0.12), Ningxia (0.11), Henan (0.11), Jiangsu (0.09), Zhejiang (0.08), Guangdong (0.06), Tianjin (0.06), Beijing (0.05), Shanghai (0.02), and Shaanxi (0.00). The fragmentation of reclaimed cropland in descending order is Hunan (0.45), Fujian (0.43), Hubei (0.36), Guangxi (0.34), Beijing (0.32), Zhejiang (0.32), Shaanxi (0.30), Jiangsu (0.28), Chongqing (0.27), Jilin (0.27), Sichuan (0.26), Guangdong (0.26), Liaoning (0.26), Guizhou (0.24), Tianjin (0.23), Jiangxi (0.22), Yunnan (0.21), Shanxi (0.20), Shanghai (0.19), Anhui (0.16), Gansu (0.14), Inner Mongolia (0.14), Henan (0.14), Hainan (0.14), Hebei (0.13), Heilongjiang (0.13), Ningxia (0.13), Tibet (0.12), Shandong (0.10), Qinghai (0.08), and Xinjiang (0.04).

### 3.5. Discussion

A clear negative correlation was observed and could be used to prove that topographic conditions of reclaimed cropland were getting worse [52], with obvious adverse effects on overall agricultural output. Nearly 70% of all food produced was in the major grain-producing areas in China, including Henan, Shandong, Jiangsu, Heilongjiang, Sichuan, Anhui, Hebei, Jilin, Jiangxi, Hunan, and Hubei [54]. Most of these provinces contained more occupied cropland, which was found in relatively intact and unfragmented blocks of land, with smaller slopes and lower altitudes than reclaimed cropland. Therefore, a need exists to protect cropland in these provinces, especially the cropland with better terrain conditions. 

 Over the past decade, overall cropland area has decreased, but total grain yield has continued to rise. This can be attributed in part to advances in agronomic practices, improved farmland regulations, enhanced field management practices, and increased use of chemical fertilizers and pesticides. Nevertheless, these methods have their limits in expanding agricultural production. Even though we can enhance the per hectare yield using advanced technology, the continued loss of cropland will inevitably lead to a reduction in food yield in the near future.

 In China, development has made great contributions to providing a secure food supply. An abundance of high quality cropland has been urbanized and used to produce more economic value through industrial uses than it could have as agricultural land. In general, the developing areas had poorer natural conditions for agriculture than the developed areas. However, in a long term view, this trend will lead to instability in the primary productivity of developing areas. At the same time, the weak economic conditions in developing areas will likely result in decreased farm income, thereby deflating farmers' enthusiasm for working to increase production; this could lead to migration of farmers to cities and developed regions, which could then lead to labor shortages in areas that rely heavily on cultivation. This would have a profound impact on China's efforts to maintain a secure food supply and sustainable agricultural development.

## 4. Conclusions

As the urbanization and industrialization of China occur, and China attempts to replace this occupied cropland with newly reclaimed cropland, the topography of the most recently reclaimed cropland has been more poorly suited to farming than the topography of the occupied cropland it replaces in most provinces. In different provinces, this phenomenon is evident in attempts to grow crops on areas in which farming is limited by slope, elevation, or fragmentation. Results from nine provinces showed that occupied cropland had better terrain conditions than reclaimed cropland in terms of the factors of slope, elevation, and fragmentation.

 The area of occupied cropland was still decreasing in the periods 2000–2005, 2005–2008, and 2008–2010; reclaimed cropland area displayed the same trend. The proportion of occupied and reclaimed cropland basically remained balanced during each period, but the area of occupied cropland was much larger than that of reclaimed cropland. It is noteworthy that cropland replenishment, the replacement of occupied cropland with reclaimed cropland, was concentrated mainly in northern China instead of southern China from 2000 to 2010. Furthermore, in most cases, cropland has been taken over mainly by urbanization in the developed parts of China to accommodate a rapid increase in the gross national product. Our findings imply that the potential area available for cropland production may be limited. Cropland protection should be coordinated at a national level.

## Figures and Tables

**Table 1:** Slope rating and characteristics.

Slope	Characteristics
0–5°	Low slope, gentle incline, smooth water movement, little soil erosion, optimal slope condition for agricultural cultivation.
5–15°	Medium slope, motive force and gravity effects increased, water movement accelerated, corrosion and soil erosion increased but not severe. Better slope condition for farming.
15–25°	Abrupt slope, erosion and block movement relatively intense, soil erosion relatively severe, just able for cultivation, upper limit condition for cultivation, but farming benefit is not well.
Above 25°	Steep slope, rain erosion and block movement intensified with slope increased, strong corrosion, severe soil and water loss, soil layer get thinner and bare rocks increased, unsuitable for cultivation but well enough for developing forestry and sideline.

**Table 2:** Area of occupied and reclaimed cropland during different time periods.

Year	Cropland area (×10^4^ hm^2^)		Percentage	
	Occupation	Reclamation	Occupation	Reclamation
2000–2010	451.88	315.94	100.00%	100.00%
2000–2005	256.65	198.37	56.80%	62.79%
2005–2008	117.95	65.28	26.10%	20.66%
2008–2010	83.05	57.57	18.38%	18.22%

**Table 3:** Cropland occupation and reclamation in all provinces (excluding Hong Kong, Macau, and Taiwan) in China from 2000 to 2010.

Province	Cropland reclamation	Cropland occupation	Total	Cropland reclamation	Cropland occupation	Reclamation classification	Occupation classification
	(×10^4^ hm^2^)	(×10^4^ hm^2^)	(×10^4^ hm^2^)	Rate (%)	Rate (%)		
Xinjiang	146.56	14.87	131.69	24.93%	2.53%	1	3
Heilongjiang	38.2	16.51	21.69	2.40%	1.04%	2	3
Jilin	10.39	5.54	4.85	1.39%	0.74%	3	3
Hebei	2.94	14.71	−11.77	0.30%	1.52%	3	3
Inner Mongolia	49.27	33.64	15.63	4.39%	3.00%	2	3
Beijing	0.22	5.92	−5.7	0.45%	12.18%	3	2
Tianjin	0.32	6.43	−6.11	0.46%	9.08%	3	2
Liaoning	3.9	8.24	−4.33	0.61%	1.28%	3	3
Ningxia	5.52	14.39	−8.86	3.01%	7.84%	2	2
Shandong	10	31.24	−21.24	0.97%	3.04%	3	3
Shaanxi	3.22	24.4	−21.18	0.45%	3.43%	3	3
Shanxi	0.98	14	−13.02	0.16%	2.31%	3	3
Qinghai	1.67	1.12	0.54	2.05%	1.38%	2	3
Gansu	15.25	16.82	−1.57	2.35%	2.59%	2	3
Henan	1.88	18.05	−16.17	0.17%	1.68%	3	3
Jiangsu	0.94	37.99	−37.05	0.14%	5.48%	3	2
Tibet	0.07	0.19	−0.12	0.16%	0.41%	3	3
Shanghai	0.21	8.19	−7.99	0.47%	18.77%	3	1
Anhui	1.47	18.32	−16.85	0.18%	2.29%	3	3
Chongqing	1.55	12.11	−10.56	0.40%	3.16%	3	3
Hubei	1.21	17.62	−16.4	0.18%	2.56%	3	3
Zhejiang	2.06	26.6	−24.54	0.75%	9.62%	3	2
Sichuan	0.74	21.27	−20.53	0.06%	1.77%	3	3
Jiangxi	6.95	8.94	−2	1.56%	2.00%	3	3
Guizhou	6.79	10.96	−4.17	1.39%	2.24%	3	3
Hunan	0.37	7.81	−7.43	0.06%	1.29%	3	3
Fujian	0.64	11.38	−10.73	0.30%	5.25%	3	2
Yunnan	1.5	13.62	−12.11	0.22%	2.01%	3	3
Guangxi	0.33	4.35	−4.01	0.07%	0.85%	3	3
Guangdong	0.17	24.79	−24.63	0.04%	5.57%	3	2
Hainan	0.61	1.86	−1.26	0.69%	2.12%	3	3

**Table 4:** The comparison between the average slope of occupied and reclaimed cropland and average slope of cropland in all provinces (excluding Hong Kong, Macau, and Taiwan) in China from 2000 to 2010.

Province	Average slope of cropland (°)	Average slope of reclaimed cropland (°)	Average slope of occupied cropland (°)
Xinjiang	0.6	0.39	0.61
Heilongjiang	0.24	0.29	0.41
Jilin	0.34	0.31	0.35
Hebei	0.81	0.26	0.37
Inner Mongolia	0.4	0.36	0.39
Beijing	1.41	0.39	0.34
Tianjin	0.11	0	0.03
Liaoning	0.78	0.68	0.58
Ningxia	0.69	0.45	0.45
Shandong	0.4	0.04	0.3
Shaanxi	1.92	0.94	1.17
Shanxi	1.18	0.52	0.85
Qinghai	2.38	1.52	1.72
Gansu	1.8	0.94	2.17
Henan	0.47	0.05	0.27
Jiangsu	0.07	0.04	0.06
Tibet	5.36	0.2	2.23
Shanghai	0.02	0.03	0.02
Anhui	0.61	0.16	0.23
Chongqing	3.43	4.19	3.98
Hubei	1.7	1.36	0.84
Zhejiang	2.32	3.26	0.92
Sichuan	3.5	7.47	3.23
Jiangxi	1.36	0.92	0.55
Guizhou	1.96	1.57	2.18
Hunan	1.38	0.91	1.01
Fujian	2.79	2.64	1.42
Yunnan	3.86	3.49	2.36
Guangxi	1.27	0.95	0.44
Guangdong	1.46	0.2	0.45
Hainan	0.73	1.05	0.75

**Table 5:** Comparison between the average elevation of occupied and reclaimed cropland and average elevation of cropland in all provinces (excluding Hong Kong, Macau, and Taiwan) in China from 2000 to 2010.

Province	Average altitude of cropland (m)	Average altitude of reclaimed cropland (m)	Average altitude of occupied cropland (m)
Xinjiang	1045.72	926.98	979.67
Heilongjiang	238.16	241.93	297.52
Jilin	333.05	365.49	303.89
Hebei	500.13	659.02	164.98
Inner Mongolia	916.9	858.39	1100.82
Beijing	276.42	117.06	75.65
Tianjin	14.22	3.82	7.05
Liaoning	239.4	234.49	179.81
Ningxia	1669.91	1463.49	1544.77
Shandong	103.03	17.49	69.8
Shaanxi	1062.7	1051.1	946.73
Shanxi	1150.19	770.26	1138.74
Qinghai	2973.53	3370.92	2625.24
Gansu	1847.06	1639.8	1794.49
Henan	209.35	189.59	160.33
Jiangsu	28.48	19.77	30.79
Tibet	3741.97	4411.76	3960.32
Shanghai	5.09	8.76	4.67
Anhui	98.46	53.18	65.91
Chongqing	701.87	682.86	725.7
Hubei	372.76	339.31	188.64
Zhejiang	238.77	358.55	105.36
Sichuan	1141.2	2001.63	1097.55
Jiangxi	237.2	197.12	130.88
Guizhou	1120.08	1078.74	1146.77
Hunan	337.89	250.27	259.09
Fujian	437.4	505.32	201.52
Yunnan	1736.01	1486.84	1700.53
Guangxi	353.08	358.9	259.39
Guangdong	178.94	69.14	67.58
Hainan	119.69	78.36	99.79
